# Image-Based Phenotyping of Flowering Intensity in Cool-Season Crops

**DOI:** 10.3390/s20051450

**Published:** 2020-03-06

**Authors:** Chongyuan Zhang, Wilson A. Craine, Rebecca J. McGee, George J. Vandemark, James B. Davis, Jack Brown, Scot H. Hulbert, Sindhuja Sankaran

**Affiliations:** 1 Department of Biological Systems Engineering, Washington State University, Pullman, WA 99164, USA; chongyuan.zhang@wsu.edu; 2 Department of Crop and Soil Science, Washington State University, Pullman, WA 99164, USA; wilson.craine@wsu.edu (W.A.C.); scot_hulbert@wsu.edu (S.H.H.); 3 USDA-ARS, Grain Legume Genetics and Physiology Research, Washington State University, Pullman, WA 99164, USA; rebecca.mcgee@usda.gov (R.J.M.); george.vandemark@usda.gov (G.J.V.); 4 Department of Plant Sciences, University of Idaho, Moscow, UI 83844, USA; jdavis@uidaho.edu (J.B.D.); jbrown@uidaho.edu (J.B.)

**Keywords:** image processing, multispectral imaging, phenomics, plant breeding, UAV

## Abstract

The timing and duration of flowering are key agronomic traits that are often associated with the ability of a variety to escape abiotic stress such as heat and drought. Flowering information is valuable in both plant breeding and agricultural production management. Visual assessment, the standard protocol used for phenotyping flowering, is a low-throughput and subjective method. In this study, we evaluated multiple imaging sensors (RGB and multiple multispectral cameras), image resolution (proximal/remote sensing at 1.6 to 30 m above ground level/AGL), and image processing (standard and unsupervised learning) techniques in monitoring flowering intensity of four cool-season crops (canola, camelina, chickpea, and pea) to enhance the accuracy and efficiency in quantifying flowering traits. The features (flower area, percentage of flower area with respect to canopy area) extracted from proximal (1.6–2.2 m AGL) RGB and multispectral (with near infrared, green and blue band) image data were strongly correlated (*r* up to 0.89) with visual rating scores, especially in pea and canola. The features extracted from unmanned aerial vehicle integrated RGB image data (15–30 m AGL) could also accurately detect and quantify large flowers of winter canola (*r* up to 0.84), spring canola (*r* up to 0.72), and pea (*r* up to 0.72), but not camelina or chickpea flowers. When standard image processing using thresholds and unsupervised machine learning such as k-means clustering were utilized for flower detection and feature extraction, the results were comparable. In general, for applicability of imaging for flower detection, it is recommended that the image data resolution (i.e., ground sampling distance) is at least 2–3 times smaller than that of the flower size. Overall, this study demonstrates the feasibility of utilizing imaging for monitoring flowering intensity in multiple varieties of evaluated crops.

## 1. Introduction

Flowering time and duration are two important traits in plant physiology, breeding, and agricultural production. The appearance of the flowers highlights the transition of the crop from vegetative to reproductive developmental stages, and their timing has an effect on seed and fruit yield [1]. Flowering time is consistently evaluated to help identify breeding lines and cultivars that are adapted to local environment conditions. Cool season crops are frequently selected based on flowering duration for stress (heat and drought) avoidance, especially since temperature can have an effect on anthesis and fertilization. In general, identifying and selecting for optimal flowering time can help plants avoid abiotic stresses, such as frost, drought, heat, and rain [2,3,4,5]. Some abiotic stresses can compromise yield and quality directly, for example, through male sterility or pre-harvest sprouting. For instance, heat stress (30 °C for 3 days) and water stress (leaf water potential of −2.54 Mpa) in wheat can lead to partial or complete male sterility [6]. In addition, flowering duration is another important factor, especially in commercial farm and orchard production. It is critical that seeds or fruits mature uniformly and within a short time frame, so that the grower can harvest seeds mechanically or hand-pick fruits in a single operation.

High-throughput phenotyping (HTP) techniques, including sensors [7], systems [8], and software [8,9], have been developed in recent years to phenotype plant traits. New techniques are needed to phenotype plant traits rapidly and accurately, and assist physiologists, breeders, and agronomists in objective decision-making. The traits that have been evaluated using such HTP methods include early crop vigor, plant or tree height, root architecture, size and color of seeds, etc. [10,11,12,13,14].

Flower detection has been tested on plant species such as cereals (rice, wheat, and corn), legumes (soybean), tree fruits (apple and citrus), oilseeds (lesquerella and canola), fibers (cotton), and ornamentals (rose) [15,16,17,18,19,20,21,22,23,24]. In literature, image-based detection of tangerine and lesquerella flowers showed good relationship between manual and image-based detection (R^2^ = 0.91 and 0.87–0.91, respectively) [19,20]. Similarly, rose flower were detected with an accuracy of 83.3% accuracy [22]. In reported studies, different color spaces (RGB, hue-saturation-intensity (HSI), or vegetation index extracted from RGB color space) and morphological features (e.g., circular shape) have been used [19,21,22,23]. In addition, texture features and machine learning algorithms, such as convolutional neural network (CNN) and support vector machine (SVM), have been applied to increase flower detection accuracy [15,16,18,23,24]. Sadeghi-Tehran et al. [16] used scale invariant feature transformation, local linear constraint coding, and spatial pyramid matching to extract low-and mid-level features, and applied SVM to classify images to determine heading and flowering stages of six varieties of wheat. The reported accuracy was 95.2–99.6% for heading and 85.5% for flower detection.

The reported flower detection studies have demonstrated the feasibility of detecting flowering using imaging in multiple crops. However, in most cases, these studies have focused on limited varieties and/or proximal sensing using RGB digital camera. Similar studies on cool season crops such as legumes are lacking. Therefore, the overall goal in this study was to evaluate the potential of imaging for monitoring flowering intensity in four cool-season crops‒canola, camelina, pea, and chickpea—within the context of plant breeding programs. The specific objectives of this study were to:

(1) Compare the performance of the RGB and multispectral sensors in flower detection and monitoring. The hypothesis behind this objective was that the multispectral sensor can capture reflectance in near-infrared regions, which will assist in efficient image processing, especially during flower segmentation and noise removal, compared to RGB image.
(2) Identify the impact of spatial resolution (proximal and remote sensing) on image-based flower detection accuracy. The hypothesis behind this objective was that the image resolution will affect flower detection based on the flower size and it is necessary to understand the impact of resolution on the detection accuracy.
(3) Evaluate thresholding-based method and un-supervised machine learning (k-means clustering) technique for flower detection (in pea and canola). The hypothesis behind this objective was that the un-supervised machine learning technique will provide superior performance than standard image processing methods.
(4) Evaluate the relationship between flower intensity and crop yield. The hypothesis behind this objective was that crop yield will be positively correlated with flower intensity.

## 2. Materials

### 2.1. Field Experiments and Visual Ratings

The study involved four cool-season crop breeding trials: canola (*Brassica napus* L. for winter canola, and *B. napus* L. and *B. rapa* L. for spring canola), camelina (*Camelina sativa* L.), pea (*Pisum sativum* L.), and chickpea (*Cicer arietinum* L.). Details on each experiment are summarized in Table 1. All crop trials were planted using a randomized complete block design. Image and visual rating data acquisition were acquired on the same date at three time points, referred as early, mid, and late flowering stages in all crops, with two exceptions. Early flowering stage data acquisition for spring canola (using proximal and remote sensing), and pea and chickpea (using remote sensing) was not conducted due to adverse (cloudy and/or windy) weather conditions. Camelina was planted on three different dates, and it resulted in different flowering intensities on the same date (Supplementary Materials Figure S1). Flowering stage labels for camelina were based on the plots planted on 7 May 2018. The visual ratings of flowering were recorded based on percentage of flowering plants (labeled as 0–10 for canola and camelina, and 0–100% for pea and chickpea) by the same researcher for each crop at each time point. The visual evaluation of flowers is often the standard procedure that the breeders/researchers utilize for phenotyping flowering trait [25,26,27].

### 2.2. Data Acquisition Using Sensing Techniques

**Figure 1:**
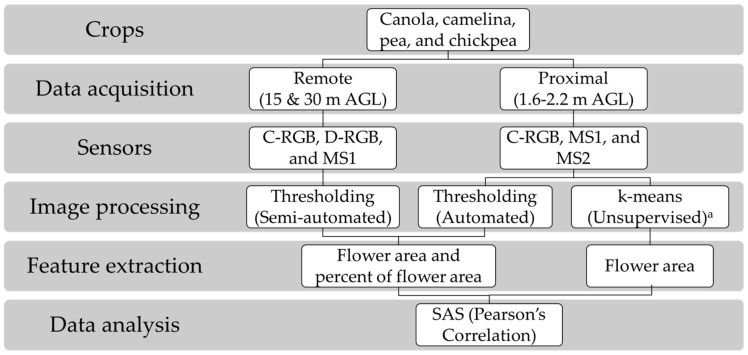
Workflow of monitoring flowering intensity in cool-season crops using sensing techniques. AGL: above ground level; C-RGB camera: canon digital/RGB camera (PowerShot SX260 HS); D-RGB: RGB camera of DJI Phantom 4 Pro; MS1 and MS2 cameras: modified digital cameras with one channel acquiring near infrared spectra of 680–800 nm and 800–900 nm, respectively; ^a^: Flowering detection using k-means clustering was tested in winter canola and pea only.

Proximal (1.6–2.2 m above ground level/AGL) and remote sensing (15–30 m AGL) images of flowering were acquired using multiple sensors, which include two RGB (C-RGB and D-RGB), and two multispectral (MS1 and MS2) digital cameras. MS1 sensor had near infrared (NIR)-G-B bands, while MS2 sensor had R-G-NIR bands. The major difference between the multispectral cameras was the NIR wavelengths; MS1 sensor had NIR band close to red-edge region, while MS2 sensor had NIR band at farther NIR region. MS2 sensor was only evaluated at early flowering stage during proximal sensing for evaluation purposes. Further details on these sensors are summarized in Table 2. The workflow of monitoring flowering intensity in this study is depicted in Figure 1. During proximal sensing, sensors were mounted on top of a tripod (canola, camelina) or on an L-shaped extension pole (pea, chickpea), and were triggered manually. During proximal sensing, a small reference panel (5 cm in diameter, Spectralon^®^ Diffuse Reflectance Standards, SRS-99-020, Labsphere Inc., North Sutton, NH, USA) was mounted for radiometric correction during image processing.

Two unmanned aerial vehicles (UAVs) were used to carry sensors to acquire aerial images for all crops: ATI AgBOT (ATI Inc., Oregon City, OR, USA) and Phantom 4 Pro (DJI Inc. Los Angeles, CA, USA.). Two sensors, C-RGB and MS1, were mounted on ATI AgBOT and set to trigger automatically at 2 s interval. The RGB sensor on the DJI Phantom 4 Pro (D-RGB) was mounted on its gimbal during data acquisition. Based on findings in our previous study in canola [29], data acquisition at 30 m AGL resulted in decent accuracies. In addition to 30 m, data acquisition at 15 m AGL was utilized to increase accuracy and avoid spectral mixing. Flight paths were planned with two mission planning software packages, Mission Planner (http://ardupilot.org/planner) for ATI AgBOT and Pix4Dcapture (Pix4D Inc., San Francisco, CA, USA) for DJI Phantom 4 Pro, such that the imaging system acquired images with 80% front and 70% side overlap. The speed of the two UAVs was set at 3 or 2 m/s to accommodate for the low flight altitudes. A 30 × 30 cm reference panel (Spectralon^®^ Diffuse Reflectance Targets, SRS-99-120, Labsphere Inc., North Sutton, NH, USA) was used for radiometric calibration.

### 2.3. Image Processing and Feature Extraction

**Figure 2:**
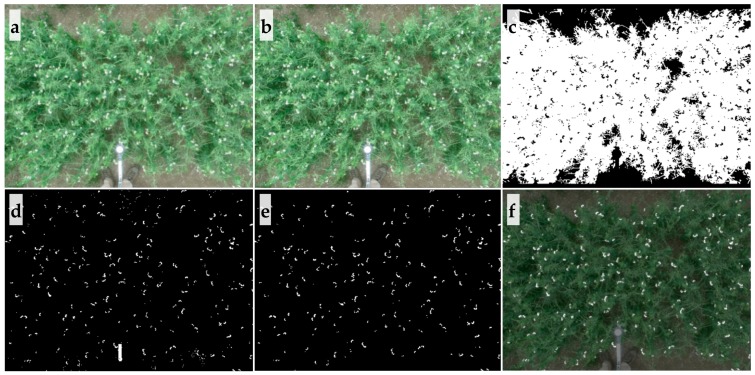
Procedure of image processing for pea. (**a**) Raw image; (**b**) radiometrically corrected image; (**c**) mask image for canopy; (**d**) mask for potential flowers with noises; (**e**) mask for potential flowers with noises removed; (**f**) overlapping of original image and noise-free flower mask.

Image processing algorithms used to analyze data were designed and programmed in MATLAB (MathWorks Inc., Natick, MA, USA). Algorithms analyzed proximal images (1.6–2.2 m AGL) in a completely automated manner. Image thresholds varied based on crop and sensor bands for segmenting canopy and flower within an image. Sample image analysis in pea is explained below. Firstly, the raw image (Figure 2a) was radiometrically corrected using the reference panel. Using the corrected image (Figure 2b), the canopy in each image (Figure 2c) was separated, and prepared for noise removal (resulting from wheat straw, branches, soil, senescent leaves and stems, artificial objects as cloth, pole, etc.) and calculation of percentage of flowers. Images were converted from RGB color space to CIE L*a*b*color space [30] to separate the canopy. A threshold of a* channel (a* < −13, optimized threshold for all pea images) could separate pea canopy from background.

**Figure 3:**
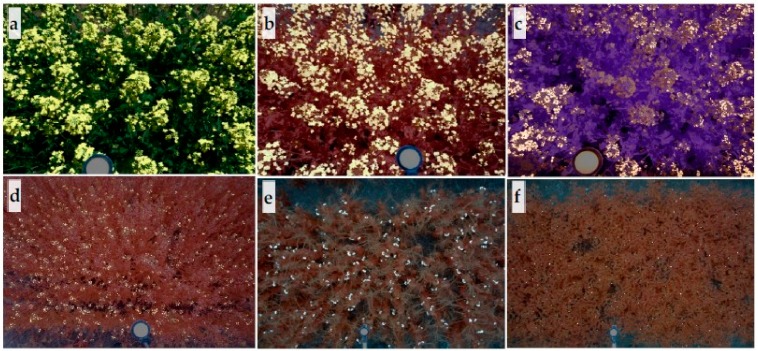
Examples of processed images with flower highlighted. Overlapped flower mask and original images derived from (**a**) C-RGB, (**b**) MS1, and (**c**) MS2 sensors for winter canola, and MS1 sensor image for (**d**) camelina, (**e**) pea, and (**f**) chickpea. Flowers are highlighted by white. Undetected winter canola flowers under shadow can be seen in (**a**) or (**c**).

After detecting the canopy, potential flowers (Figure 2d) were separated using thresholds (C-RGB sensor images: R, G, B > 200, G – R ≤ 20, valid for all pea images as well). Thresholds used for separating the canopy and flowers in images from different sensors and crops were selected according to the spectral reflectance of canopy and flowers, which was optimized based on sample images collected at different time points (Supplementary Materials Table S1). The thresholds were visually assessed and compared with original images for final selection. Upon flower detection, noises from wheat straw, branches, soil, etc. were removed using morphological operations (e.g., area, major axis to minor axis ratio, solidity) and based on neighbor connectivity (Figure 2e). For example, some noise from wheat straw or soil showed reflectance similar to that of white pea and chickpea flowers in RGB images, but were absent within the plant canopy. The logic in the algorithm scanned the peripheral pixels of candidate flowers and removed those that had no neighbor pixels as canopy. Finally, detected flowers were overlapped with the original images for quality inspection, as shown in Figure 2f and Figure 3.

**Figure 4:**
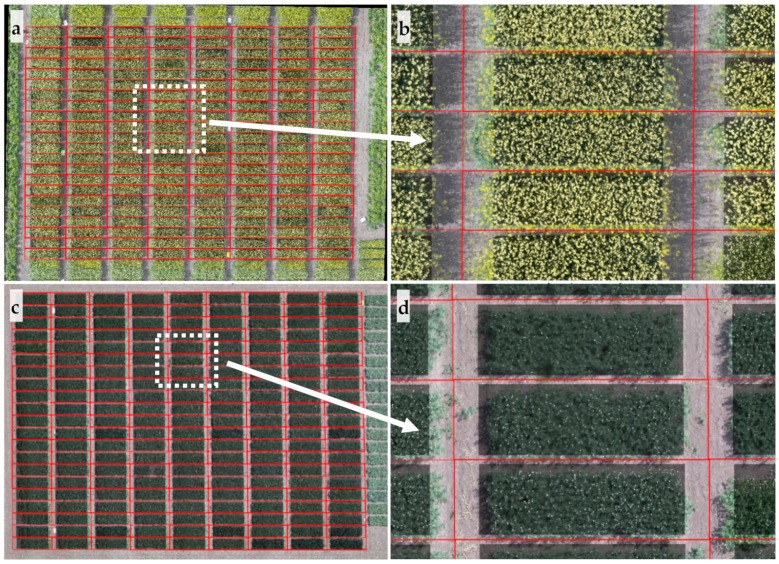
Results of flower extraction from aerial images of spring canola and pea. Separated individual plots for spring canola and pea are outlined by red rectangles as shown in (**a**) and (**c**), respectively; regions of interest (plot with four edges removed) were dimmed while flowers were highlighted in white as shown in (**b**) and (**d**); (**b**) and (**d**) are zoom-in images of white-highlighted areas of (**a**) and (**c**), respectively.

In regard to remote sensing data (15–30 m AGL), images were processed semi-automatically with initial inputs from the user. First, aerial images were processed using software to create orthomosaic images covering the entire experimental fields. Images from the D-RGB sensor were stitched in Pix4Dmapper (Pix4D Inc., San Francisco, CA, USA); while images without geotag data (C-RGB and MS1 sensors) were processed in Agisoft PhotoScan Professional (Agisoft LLC, St. Petersburg, Russia). Following orthomosaic image generation, the image was imported and further processed (rotate, crop, and radiometrically calibrate using reference panel) in MATLAB. The canopy and flowers were segmented prior to extracting flower features from individual plots. The procedures used to separate canopy and flowers from orthomosaic images were similar to those described for proximal images, with some changes in thresholds. After these steps, the four corners of the field containing all the plots were identified manually in the orthomosaic images. With the field location information, the algorithms automatically separated individual plots using interpolated location of plots and extracted flower features from the central part of each plot, as illustrated in Figure 4. Images derived from the D-RGB sensor for all crops were analyzed; however, images from the C-RGB and MS1 sensors for winter and spring canola, and pea were considered for further analysis due to the larger flowers and higher image quality. Similarly, overlapping original image and noise-free flower mask images were exported for quality inspection. Regardless of the sensing methods (proximal and remote), two features, flower area (in pixels) and percentage of flowers (ratio of flower area to canopy area that includes flowers), were extracted from images. Data were exported at the end of image processing step for statistical analysis.

Data from one data acquisition date for winter canola and pea were processed to test the efficiency of unsupervised machine learning (k-means clustering) for flowering monitoring. The algorithms separated pixels in different clusters through k-means clustering and identified flower clusters based on color (white or yellow). In this study, the k-means clustering was implemented in MATLAB using set maximum output clusters as 6 (k = 6), squared Euclidean distance as distance calculation method (each centroid is the mean of the points in that cluster), and three replicates to repeat clustering. Other parameters used in the k-means clustering were default settings in MATLAB, which include maximum iteration of 100, ‘singleton’ action where a new cluster consisting of the one point furthest from its centroid is created, and initial cluster centroid position method of ‘Plus’ in MATLAB (that selects k seeds by implementing the k-means++ algorithm for cluster center initialization [31]; the k-means++ algorithm uses an heuristic to find centroid seeds for k-means clustering, which improves the running time of Lloyd’s algorithm and quality of final solution). More details can be found at: https://www.mathworks.com/help/stats/kmeans.html#bueftl4-1. A set of thresholds were used to automatically identify the flower clusters through color, for example R, G, B > 0.75 (radiance of image were rescaled to the range of 0 to 1) for pea, and R > 0.75, G > 0.75, B < 0.25 or R > 0.45, G > 0.55, B < 0.12 for canola. Flower area was the only feature extracted from these images using k-means clustering algorithm.

### 2.4. Statistical Analysis

The relationship of flower features and ground truth data (visual rating of flowering and seed yield at harvest) were analyzed using Pearson’s correlation analysis in SAS University Edition (3.7, SAS Institute, Cary, NC, USA).

## 3. Results

### 3.1. Flower Detection Using RGB and Multispectral Sensors

Three sensors (C-RGB, MS1, and MS2) were used for detecting flowers using proximal sensing platforms (1.6–2.2 m AGL). The performance of these sensors in detecting flowers and its intensity were assessed based on correlation between visual rating scores and flower features extracted using thresholding method, as summarized in Table 3. C-RGB and MS1 sensors showed good performance in flower and intensity detection across all four crops in most sampling dates. The results indicated that both C-RGB and MS1 images could detect flowering of winter canola, spring canola, and pea with medium to high correlation coefficients (*r* = 0.56–0.89, *p* < 0.001) using flower area or percentage of flowers. The MS1 images were better for detecting camelina flowering using flower area, while the C-RGB images were more precise in detecting chickpea flowering at early and mid-flowering stage. In most cases, the features extracted from MS2 images did not out-perform C-RGB or MS1 images, especially for pea and camelina flowers. However, MS2 sensor showed substantially poorer performance in detecting winter canola and chickpea flowers.

**Figure 5:**
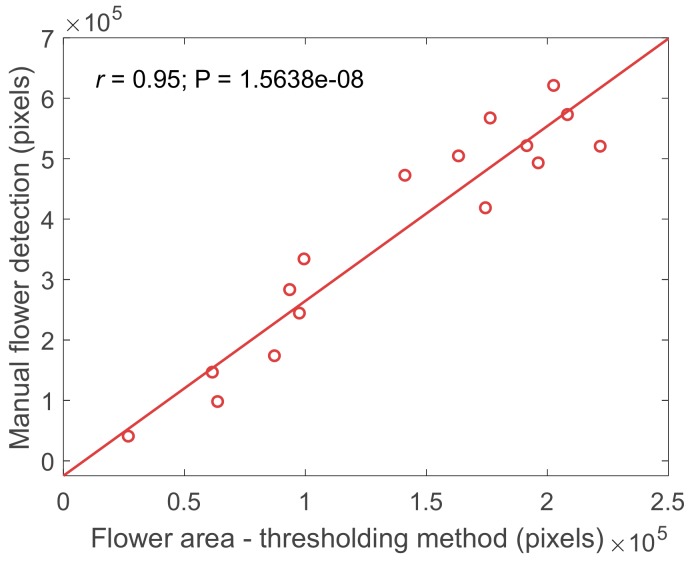
Correlation between flowers detected by thresholding method and manual identification.

At peak bloom (middle flowering stage for canola and late flowering stage for pea and chickpea), the accuracy in monitoring flowering intensity using imaging technique decreased. This could be because visual ratings were based on percentage of flowering plants, rather than flower area. At 100% bloom, the size of the flower area may increase, while number of flowering plants remains constant. Moreover, the ground reference data of visual scores in the field is also subjective. Therefore, to verify the accuracy of the flower detection technique based on thresholding, pea flowers from 16 images acquired during peak bloom stage were manually marked and used as ground truth data. The *r* value between flowers detected by thresholding and manually identified method from images was very high (*r* = 0.95, *p* < 0.001), as shown in Figure 5, compared with *r* between flowers detected based on thresholding and visual rating (r = 0.58, *p* < 0.001). This highlights the benefit of imaging over visual rating scores. Compared to thresholding method, manual identification can detect flowers of larger area and those flowers in shadow or a cluster. During image processing, clustered flowers with a high major-axis-to-minor-axis ratio were eliminated as noise (wheat straw from previous year trial had similar reflectance spectrum as pea flower, but with high major-axis-to-minor-axis ratio). Nevertheless, these results indicate that the relative abundance of flowers can easily be detected using a thresholding method, even during peak bloom. In summary, C-RGB and MS1 sensors were better at detecting flowering in the four crops than MS2 sensor, and in general, the C-RGB sensor results were better than MS1 sensor results.

The ability of sensors (D-RGB on UAV DJI Phantom 4 Pro, C-RGB, and MS1) mounted on unmanned aerial vehicles (15 m and 30 m AGL) to detect flowers was dictated by the color and size of flowers, as shown in Table 4. The three sensors resulted in similar *r* values, when imaging the larger flowers of winter canola (*r* up to 0.84, *p* < 0.001) and spring canola (*r* up to 0.77, *p* < 0.001). In contrast, the ability of the sensors to detect the large white pea flowers varied. The correlation of flower features of pea derived from the D-RGB sensor with visual rating scores at mid and late flowering period were medium to high (*r* = 0.57–0.72) and low (*r* = 0.31–0.39), respectively. The dispersion of image data from the MS1 sensor (*r* = 0.28–0.58, *p* < 0.001) were higher than those from the D-RGB sensors. For camelina and chickpea, which have relatively small flowers, no sensor demonstrated significant or meaningful correlations, except for camelina flower area at early flowering period. In general, the D-RGB sensor (~20 megapixels or MP) was better for detecting large flowers and flowering intensities, while C-RGB sensor with ~12 MP and MS1 sensor with 12–20 MP showed potential in detecting large and distinctive flowers (e.g., pea, canola).

Overall, it is feasible to monitor flowering intensity using RGB and MS1 sensors. RGB sensor demonstrated the slightly better performance in flower monitoring, followed by MS1 sensor, during proximal and remote sensing.

### 3.2. Impact of Spatial Resolution on Flower Detection

The altitude of data acquisition affects image resolution and therefore the accuracy of flower detection during image analysis. In sensing techniques, ground sample distance (GSD) refers to the physical distance between two-pixel centers as measured on the ground. Namely, smaller GSD refers to higher image resolution and higher probability of differentiating small objects (flowers in this study) from the background. In general, the optimal data acquisition altitude should be such that the GSD is smaller or equal to the size of target object (flower) that needs to be differentiated from the background. In this study, the GSD was 0.6–0.7, 4–5, and 8–10 mm for data acquired at 1.6–2.2, 15, and 30 m AGL. Thus, the optimal data acquisition altitude for flower monitoring was dependent on the size of flowers. When comparing the effect of image resolution using two sensors (C-RGB in proximal sensing and D-RGB sensors in remote sensing) for winter canola, the data acquired even at 30 m AGL showed good relationship between image features and visual rating scores (*r* = 0.75–0.84) as those derived from 1.6 m AGL data (*r* = 0.75–0.82). However, for spring canola and pea, the accuracy of monitoring flowering generally decreased as the resolution of image data decreased (when altitude increased from 1.6–2.2 m to 30 m AGL). On the other hand, the effect of image resolution on monitoring flowering for small flowered plants was more dramatic. Positive correlations were observed between visual ratings and flower features derived from proximal sensing for camelina and chickpea (Table 3 and Table 4). However, low correlations were found between visual ratings and flower features derived from remote sensing data. Only flower area derived from remote sensing data for camelina at the early flowering period was correlated positively and significantly with visual ratings. The results indicated that proximal or very low altitude (< 15 m AGL) sensing are more suitable for monitoring small flowers, such as camelina and chickpea, while altitude up to 30 m AGL can be used in evaluating the flowering intensity of large flowers.

### 3.3. Machine Learning for Flowering Detection

**Figure 6:**
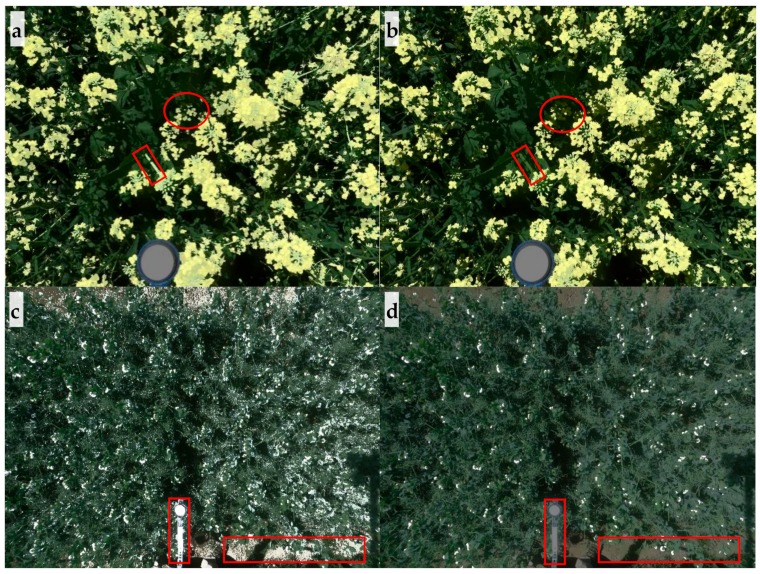
Detection of winter canola and pea flowers using by (**a**) and (**c**) k-means clustering and (**b**) and (**d**) thresholding. As examples, rectangles highlight the noise detected by k-means clustering while ovals highlight the flowers missed by thresholding.

**Figure 7:**
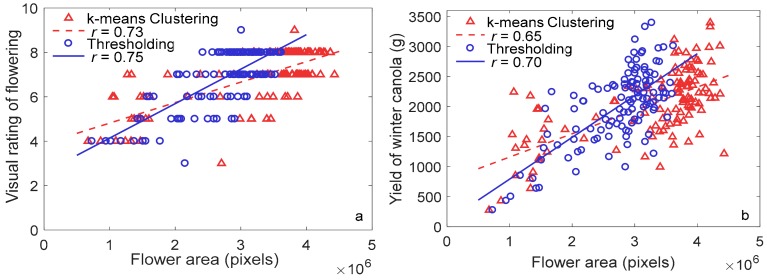
Relationship between flower area derived using k-means clustering and thresholding methods with (**a**) visual rating scores and (**b**) yield.

The effect of k-means clustering on flowering detection differed based on the color of flowers, evidenced by results from winter canola and pea. The k-means clustering detected yellow winter canola flowers under both sunlight and shadow accurately; in addition, the method also classified some stems as flowers due to similarity in spectral reflectance (Figure 6a). However, thresholding did not capture stems and flowers under shadow, as shown in Figure 6b. Moderate correlation coefficients between flower area derived from k-means clustering and ground truth (visual rating scores and yield) were found (*r* of 0.73 and 0.65, respectively; *p* < 0.0001) for winter canola. The correlation coefficients were comparable to those derived from thresholding method, as shown in Figure 7.

In contrast, k-means clustering did not correctly detect white pea flowers. For pea, the thresholding removed non-flower pixels properly, as shown in Figure 6c,d; however, k-means clustering incorrectly classified parts of leaves, extension pole, soils, and stems that were light colored or pale colored in the same cluster as flowers. Therefore, correlation analysis was not performed for pea flowering detection using k-means clustering. Although k-means clustering and thresholding were comparable in detecting flowers for canola, the amount of time required to develop the algorithm and process the images differed significantly. Development of k-means clustering-based algorithm was relatively easy and quick; while thresholding took more time to optimize thresholds to separate canopy and flowers, and develop a noise removal algorithm based on morphological operations and neighbor connectivity. However, the k-means clustering took more time than thresholding for image processing (e.g., 188.4 s vs. 5.5 s per image for winter canola using the same computer). Nevertheless, these results indicate that k-means clustering may be useful to detect flowers with a color, such as yellow, that is significantly different from the background colors.

### 3.4. Relationship between Flower Features and Seed Yield

A strong relationship between image features of flowering and seed yield was found only in winter canola. Results for canola (winter and spring), chickpea, and pea are presented in Supplementary Materials Table S2. Camelina data were not analyzed, as the data was comprised of information from multiple flowering stages within each data acquisition date. Generally, correlations between yield and visual ratings or flower features were weak for spring canola (*r* up to 0.26), pea (*r* up to 0.25), and chickpea (*r* up to 0.48). However, for winter canola, most visual rating scores or flower features were significantly (*r* up to 0.84, *p* < 0.05) correlated with yield. The correlation trend (positive or negative) between yield and visual rating scores or flower features for winter canola changed over crop development stages. Until mid or peak bloom, visual rating scores or flower features were positively correlated with yield. However, at late flowering period, the correlations became negative. Compared to visual ratings and flower features from proximal sensing, flower area derived from remote sensing, regardless of altitudes, was more strongly correlated with yield at early and mid-flowering periods (*r* up to 0.84 for remote sensing, *r* up to 0.74 for proximal sensing). In short, relationship between flower features and seed yield was weak for spring canola, pea, and chickpea, but strong for winter canola, and further study is needed to depict the relationships, especially for winter canola.

## 4. Discussion

### 4.1. Sensors for Flowering Detection

The results indicate that both RGB and MS1 sensors were suitable for detecting flowering and flower intensity using image data. However, data was affected by noise and shadow. Occasionally, image noise (non-flower objects in image misclassified as flowers) was observed, especially with images derived from proximal (1.6–2.2 m AGL) sensing data. Using C-RGB and MS1 sensors, senescent leaves and stems created noise when detecting yellow flowers of canola and camelina, while wheat straw, soil, and artificial objects (e.g. cloth and pole) resulted in noise when white flowers of pea and chickpea were the target. Noise from senescent leaves or wheat straw and soil were more predominant when canopy closure did not occur at early flowering stage, exposing the soil and lower leaves that were senescing. Multispectral cameras did not demonstrate advantage in noise removal as expected. Therefore, noise removal algorithms are necessary to increase accuracy of flower detection for images from proximal sensing. In addition, shadows created by multiple layers of flowers or canopies reduced brightness of flowers, which were found in images from all cameras. Detection of flowers with lower brightness was challenging without introducing noise from stems or soil.

In addition to visible and near-infrared spectra, ultraviolet spectrum can also be utilized for flower detection. Some flowers, such as those of crucifers [32], absorb or reflect ultraviolet (UV) light to make themselves more attractive to pollinators (e.g., bees and butterflies) that have UV (∼350 nm), blue (∼440 nm) and green (∼530 nm) receptors for color vision [33,34]. Flavonoids and de-aromatized isoprenylated phloroglucinols are reported to absorb UV light and create floral patterns or nectar guides that are visible in UV light and used to attract insects for pollination [35,36,37]. Such characteristics of certain flowers under UV light can be utilized for flower monitoring with the help of a UV camera. Two potential challenges that the user needs to aware of are: low intensity of UV light (as UV light only accounts for 3–5% [38] of solar radiation reaching the Earth’s surface) and differences in species-specific floral patterns in UV light [32].

### 4.2. Role of Spatial Resolution and Auxiliaries during Data Acquisition

The results indicated that large flowers (e.g., canola, pea) can be monitored up to 30 m AGL, while small flowers should be monitored with lower altitude (< 15 m AGL) that lead to high image resolution. Compared with altitudes (> 60 m AGL) used for monitoring others crop features (e.g., canopy area and vigor), lower altitudes must be used for flower monitoring (≤ 30 m) [29]. The optimum altitude can be determined based on size of flowers, with possibly higher altitudes for larger flowers. GSDs for D-RGB camera (20.0 MP) were 4 and 8 mm at 15 and 30 m AGL, respectively. The flower size of canola and pea were 2–3 times larger than GSDs at 15 and 30 m AGL, while the flower size of camelina and chickpea were no more than 2 times larger than GSDs at 15 and 30 m AGLs. Larger canola and pea flowers contributed to greater accuracy of flower detection and allowed monitoring up to 30 m AGL. In contrast, no useful data were obtained for small flowers, especially chickpea flowers, when using remote sensing platforms. This was because when the GSD was close to the size of flowers and some flowers fell between or among pixels, rather than within pixels, it would result in spectral mixing (between flowers and background objects) and reduce the accuracy of flowering detection. Therefore, when using remote sensing techniques for flowering monitoring, it is recommended to select the correct image resolution (or altitude) to ensure GSD is at least 2–3 times smaller than the size of flowers.

In addition to the altitude of flight, proper auxiliaries, such as a GPS unit and a gimbal, are also important to ensure the usefulness of aerial data. Agricultural land is homogeneous (or similar in terms of color and texture) and images without geotag information can be challenging to stitch for creating orthomosaic images. GPS information should be recorded during image acquisition via the UAV or external GPS unit [24]. Use of efficient gimbal that compensates for vibration and flight movement is also recommended to increase the quality of the image data collected for flowering detection.

### 4.3. Methods of Flower Detection

Different image processing methods have been evaluated for flower detection in several studies, as summarized in Table 5. In this study, two image processing methods, thresholding and k-means clustering, were evaluated. The method of k-means clustering, an unsupervised machine learning method, is easy to develop and required little optimization (e.g., number of clusters) and training. The k-means clustering worked well only for canola flowers as it has a distinct color and stands out from the background. In contrast, the thresholding method was efficient in detecting flowers from all four crops and was able to remove noise from the background. When thresholds are optimized, the thresholding method is a high-throughput way of accurately analyzing images. On other hand, supervised methods, such as SVM and CNN, require substantial time to develop annotated data, and to develop and optimize the model. Typically, these methods also require big data for model development. Supervised methods can especially be useful for flower detection in crops without conspicuous flowers, such as cereal crops (Table 5). Nevertheless, methods used for flower detection can be selected based on the appearance and color of flowers and desired accuracy.

### 4.4. Flower-Based Yield Estimation

The negative correlation between yield and visual rating scores or flower features for winter canola occurred during pod fill (late flowering). Seeds formed at early or mid-flowering stages have a longer pod filling period to accumulate photosynthate that results in higher yield, compared to those formed at late flowering stage. It has been reported that the duration of grain fill is positively correlated with yield in cereal crops [41,42,43]. Besides grain filling period, grain filling rate [41], and the percentage of fertile flowers also are critical components of yield. The association of the flowering stage and duration with yield is a complex phenomenon, which needs to be further investigated.

### 4.5. Improving Accuracy of Flower Detection and Implication of Flower Monitoring Using HTP Techniques

Flower detection using thresholding or machine learning methods (supervised or unsupervised) experiences similar challenges, and future research can focus on improving accuracy for flower detection through illumination compensation and noise removal. One challenge of flower detection, or phenotyping other plant traits using optical sensing, is uneven illumination due to shadow, as found in this study and Xu et al. [24]. Depending on how broad or narrow the thresholds were, uneven illumination led to overestimation or underestimation of flowering by incorrectly including noise or excluding flowers. To increase accuracy, shadow detection and illumination compensation methods [44,45,46] may be used to mediate the issue of uneven illumination. Another challenge in detecting flowers is noise from soil, wheat straw, leaves, and artificial objects. For example, leaves and soil that strongly reflect sunlight, shown in Figure 6c, are difficult to remove based only on color. Morphological operations and neighbor connectivity were used in this study to remove most noise successfully. To remove noise with shape and connectivity similar to those of flowers, height information [24] (from crop surface models) can be used.

The standard method of evaluating flowering is based on visual rating scores. Proximal (with potential integration with field platforms) or remote sensing allows high frequency and throughput in flowering data acquisition. In addition, image processing analyzes images using quantitative and constant parameters, such as thresholds and area for noise removal, and evaluates flowering in non-subjective and quantitative ways. In this study, these methods were found to be useful in estimating flowering intensity. With high temporal and spatial resolution image data, flowering dynamics (or flowering curve) for each genotype can be monitored efficiently and accurately. Flower features can be computed based on the flowering dynamics, such as 50% flowering, and flowering time and duration. Breeding lines can be selected based on time of flowering under local conditions. For example, early-flowering and short duration of flowering varieties of pea and canola are more suitable for regions with Mediterranean climate, where crops have to produce seeds before the onset of terminal heat and drought stress [47,48]. Monitoring flowering with sensors can detect flowering responses or behaviors triggered by stressors, which can assist in the study of flowering physiology and genes associated with flowering. Flowering data can be useful in estimating yield in tree fruit production and guide agricultural practices, such as flower thinning [17].

## 5. Conclusions

The study demonstrates the development and evaluation of image based HTP techniques for quantifying flowering intensity of multiple varieties in canola, camelina, pea, and chickpea. The study used digital visible (RGB) and multispectral (NIR-G-B or R-G-NIR) sensors to monitor flowering intensity. The results indicated moderate to high correlation coefficients between flower features and visual rating scores were achieved in all four crops using proximal sensing (*r* up to 0.89), and in winter and spring canola and pea using remote sensing (*r* up to 0.84). The unsupervised k-means clustering was effective in detecting canola flowers, but was not effective in eliminating noise in pea flower images. Both thresholding and k-means clustering methods were affected by noise and uneven illumination during flower detection. Significant and strong correlation between image features of flowering and seed yield was found in winter canola (*r* up to 0.84), but weak correlation in other crops. Overall, the results of phenotyping flowering intensity in multiple varieties of different crops using image-based techniques are promising; such techniques have the potential of assisting in plant breeding and crop production.

## Figures and Tables

**Table 1:** Summary of experiments for four cool-season crops in breeding programs.

Crops	Winter Canola	Spring Canola	Camelina	Pea	Chickpea
Flower size (mm)	15–20 (dia.) ^a^	15–20 (dia.)	3.5–4.5 (dia.) ^b^	18–27 × 13–19 (L × W)	7–11 × 8–11 (L × W)
Location	Kambitsch Farm, ID	Kambitsch Farm, ID	Cook Farm, WA	Spillman Farm, WA	Spillman Farm, WA
Entries	30	44	12	55	21
Replicates	4	4	3 or 1 ^c^	3	3
Planting Date	27 September 2017	3 May 2018	7 and 25 May, 11 June, 2018 ^d^	5 May 2018	5 May 2018
Data acquisition (DAP)	229, 236, and 245	57 and 67	60, 74, and 80 ^e^	48, 53, and 59	48, 53, and 59

DAP: days after planting; L and W: length and width of flowers; ^a^ size in diameter; ^b^ [28]; ^c^ 3 reps for 9 genotypes or 1 rep for 3 genotypes; ^d^ Date of early, mid, and late planting dates, respectively; ^e^ DAP for early planting date.

**Table 2:** Summary of sensors used in proximal and remote sensing.

Factor	C-RGB	MS1	MS2	D-RGB
Model	Canon PowerShot SX260 HS, Canon U.S.A. Inc., Melville, NY, USA	Canon ELPH 110/160 HS, LDP LLC, Carlstadt, NJ, USA ^a^	Canon ELPH 130 HS, LDP LLC, Carlstadt, NJ, USA	Camera of DJI Phantom 4 Pro, DJI Inc., LA, CA, USA
Spectrum	Visible/R, G, B ^b^	NIR ^c^ (680–800 nm), G, B	R, B, NIR (800–900 nm)	Visible/R, G, B
Resolution (megapixels)	12.1	16.1/20.0	16.0	20.0
Focal length used (mm)	4.5	4.3/5.0	5.0	8.8
GSD ^d^ (mm, proximal)	0.6/0.7	0.6/0.5	0.6/0.8	-
GSD ^e^ (mm, remote)	5 and 10	5 and 11/4 and 7	-	4 and 8
Geotagged image	No	No	No	Yes
Application	Proximal and remote sensing	Proximal and remote sensing	Proximal sensing	Remote sensing

^a^ Canon ELPH 110 HS was used for canola and camelina; Canon ELPH 160 HS was used for pea and chickpea; ^b^ R, G, B: red, green, and blue bands; ^c^ NIR: near-infrared band; ^d^ GSD or ground sample distance for proximal data acquired at 1.6 m (canola and camelina) and 2.2 m (pea and chickpea) above ground level, respectively; ^e^ GSD for aerial data acquired at 15 and 30 m above ground level.

**Table 3:** Correlation coefficient between features extracted from proximal sensing (1.6–2.2 m AGL) data and visual rating scores on flowering.

Sensor		C-RGB			MS1			MS2
Flowering Stage		Early	Mid	Late	Early	Mid	Late	Early
Winter canola	Flower area	0.82	0.75	0.76	0.79	0.76	0.77	0.50
		***	***	***	***	***	***	***
	Flowers%	0.82	0.75	0.75	0.77	0.73	0.74	0.15
		***	***	***	***	***	***	ns
Spring canola	Flower area	na	0.62	0.81	na	0.62	0.77	na
			***	***		***	***	
	Flowers%	na	0.64	0.80	na	0.58	0.77	na
			***	***		***	***	
Camelina	Flower area	0.60	0.27	0.27	0.64	0.36	0.40	0.68
		***	ns	ns	***	*	*	***
	Flowers%	0.63	0.02	0.25	0.67	0.28	0.41	0.53
		***	ns	ns	***	ns	*	***
Pea	Flower area	0.88	0.88	0.58	0.64	0.79	0.56	0.66
		***	***	***	***	***	***	***
	Flowers%	0.86	0.89	0.58	0.63	0.80	0.56	0.65
		***	***	***	***	***	***	***
Chickpea	Flower area	0.74	0.74	0.16	0.45	0.28	0.12	0.25
		***	***	ns	***	*	ns	*
	Flowers%	0.61	0.54	0.19	0.28	0.17	0.26	0.05
		***	***	ns	*	ns	*	ns

Flower area: the area of flowers in terms of pixels; flowers% is the percentage of flowers, or the ratio of flower area to canopy area that includes flowers. na: not available. ns: statistically non-significant at the 0.05 probability level; *, **, and ***: statistically significant at 0.05, 0.01, and 0.001 probability levels, respectively.

**Table 4:** Correlation coefficient between features extracted from remote sensing data and visual rating scores on flowering.

Camera			D-RGB			C-RGB			MS1		
Flowering Stage			Early	Mid	Late	Early	Mid	Late	Early	Mid	Late
Winter canola	Flower area	15 m	0.84	0.81	0.77	0.81	0.72	0.78	0.82	0.76	0.72
			***	***	***	***	***	***	***	***	***
	Flowers%	15 m	0.82	0.80	0.82	0.80	0.71	0.77	0.82	0.72	0.73
			***	***	***	***	***	***	***	***	***
	Flower area	30 m	0.84	0.79	0.75	0.76	0.73	0.75	0.76	0.70	0.66
			***	***	***	***	***	***	***	***	***
	Flowers%	30 m	0.79	0.78	0.79	0.72	0.72	0.72	0.74	0.70	0.68
			***	***	***	***	***	***	***	***	***
Spring canola	Flower area	15 m	na	0.42	0.72	na	0.54	0.77	na	0.50	0.66
				***	***		***	***		***	***
	Flowers%	15 m	na	0.43	0.72	na	0.54	0.77	na	0.43	0.63
				***	***		***	***		***	***
	Flower area	30 m	na	0.43	0.60	na	0.41	0.71	na	0.39	0.51
				***	***		***	***		***	***
	Flowers%	30 m	na	0.46	0.61	na	0.40	0.71	na	0.40	0.49
				***	***		***	***		***	***
Camelina	Flower area	15 m	0.36	−0.03	−0.40	a	a	a	a	a	a
			*	ns	*						
	Flowers%	15 m	0.13	−0.24	−0.49	a	a	a	a	a	a
			ns	ns	**						
	Flower area	30 m	0.40	−0.002	−0.33	a	a	a	a	a	a
			**	ns	*						
	Flowers%	30 m	0.27	−0.16	−0.31	a	a	a	a	a	a
			ns	ns	ns						
Pea	Flower area	15 m	na	0.72	0.39	na	b	0.32	na	0.55	0.42
				***	***			***		***	***
	Flowers%	15 m	na	0.72	0.39	na	b	0.32	na	0.58	0.42
				***	***			***		***	***
	Flower area	30 m	na	0.57	0.31	na	b	b	na	0.55	0.28
				***	***					***	***
	Flowers%	30 m	na	0.57	0.32	na	b	b	na	0.58	0.28
				***	***					***	***
Chickpea	Flower area	15 m	na	−0.01	0.08	a	a	a	a	a	a
				ns	ns						
	Flowers%	15 m	na	−0.05	0.11	a	a	a	a	a	a
				ns	ns						
	Flower area	30 m	na	−0.21	0.14	a	a	a	a	a	a
				ns	ns						
	Flowers%	30 m	na	−0.21	0.14	a	a	a	a	a	a
				ns	ns						

Flower area: the area of flowers in terms of pixels; flowers% is the percentage of flowers, or the ratio of flower area to canopy area that includes flowers. a: data were not analyzed due to small flowers; b: data can be extracted only from a few plots due to blur and stitching issue, and correlation analysis is not meaningful. na: not available. ns: statistically non-significant at the 0.05 probability level; *, **, and ***: statistically significant at 0.05, 0.01, and 0.001 probability levels, respectively.

**Table 5:** Comparison of methods of flower detection.

Method	Thresholding	k-Means (Unsupervised)	SVM and CNN (Supervised)
Algorithm development	Fast	Very fast	Slow, due to annotation of images and model development
Input	Images	Images	SVM: color, morphological, or texture features; CNN: Images
Training data	No	No	Yes
Flower detection per image	Fast	Slow	Fast
Example	Current study and [17,20,39]	Current study and [40]	SVM in [15,16,23] CNN in [18,24]
Crops	Apple, peach, pea, lesquerella, canola, camelina, chickpea	Canola, wheat	Rice, wheat, corn, soybean, and cotton

## Data Availability

The images/data in this study are available from the corresponding author on reasonable request.

## Supplementary Materials

The following are available online at https://www.mdpi.com/1424-8220/20/5/1450/s1, Figure S1: Layout of camelina breeding trial, Table S1: Image thresholds used for canopy and flower detection and segmentation using multiple sensors in different crops, Table S2: Correlation coefficient between yield and features extracted from image data or visual rating scores.
