# Supplementary material for: Image-Based Phenotyping of Flowering Intensity in Cool-Season Crops
Source: Sensors (Basel). 2020 Mar 6;20(5):1450. doi: 10.3390/s20051450 (PMC7085647; doi:10.3390/s20051450)
Supplement: Supplementary file 1 [file sensors-20-01450-s001.pdf]

sensors

Supplementary Materials

Image-Based Phenotyping of Flowering Intensity in Cool-Season Crops

Chongyuan Zhang [1], Wilson A. Craine [2], Rebecca J. McGee [3], George J. Vandemark [3], James B. Davis [4], Jack Brown [4], Scot H. Hulbert [2], and Sindhuja Sankaran [1,*]

[1] Department of Biological Systems Engineering, Washington State University, Pullman, WA, USA 99164; chongyuan.zhang@wsu.edu (C.Z.)
[2] Department of Crop and Soil Science, Washington State University, Pullman, WA, USA 99164; wilson.craine@wsu.edu (W.A.C.); scot_hulbert@wsu.edu (S.H.H.)
[3] USDA-ARS, Grain Legume Genetics and Physiology Research, Washington State University, Pullman, WA, USA 99164; rebecca.mcgee@usda.gov (R.J.M.); george.vandemark@usda.gov (G.J.V.)
[4] Department of Plant Sciences, University of Idaho, Moscow, UI, USA 83844; jdavis@uidaho.edu (J.B.D.); jbrown@uidaho.edu (J.B.)
* Correspondence: sindhuja.sankaran@wsu.edu (S.S.)

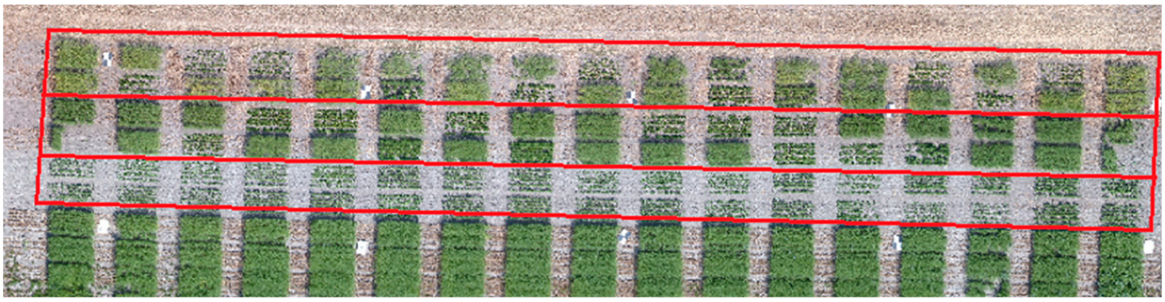

Figure S1. Layout of camelina breeding trial. Rectangles from top to bottom outline the camelina plots planted on 7 and 25 May, and 11 June, 2018, respectively.

Table S1. Image thresholds used for canopy and flower detection and segmentation using multiple sensors in different crops

**Crops**		**Winter/Spring canola**	**Camelina**	**Pea**	**Chickpea**
C-RGB	Canopy	a* < -12	a* < -15	a* < -13	a* < -18
	Flowers	0.22 > H > 0.15 & S > 0.3 & V > 0.68	0.22 > H > 0.15 & S > 0.3 & V > 0.90	R > 200 & G > 200 & B > 200 & (G - R) ≤ 20	R > 200 & G > 200 & B > 200 & (G - R) ≤ 20
MS1	Canopy	a* > 20	a* > 20	a* > -5 & b* > 0 & NIR1 > 60	a* > 0 & b* > 5 & L* > 30
	Flowers	0.22 > H > 0.10 & S > 0.2 & V > 0.50	0.28 > H > 0.10 & V > 0.30 & a* < 10	NIR1 > 160 & G > 210 & B > 220 & B - G ≤ 30 & ~canopyMask	NIR1 > 160 & G > 210 & B > 220 & B - G ≤ 30
MS2	Canopy	a* > 42	0.92 > H ≥ 0.7	(H ≥ 0.8 ∣ H < 0.06) & S ≤ 0.6 & 0.9 > V > 0.38	90 > L* > 30 & b* < 20
	Flowers	0.22 > H > 0.07 & S > 0.2 & V > 0.50	NIR2 > 50 & G > 180 & R > 220	0.15 > H ≥ 0.09 & S > 0.4 & V > 0.80	0.15 > H ≥ 0.09 & S > 0.4 & V > 0.80

L*, a*, and b* are channels for CIE L*a*b*color space, representing lightness, green-red component, and blue-yellow component; H, S, and V are channels for HSV color space, representing hue, saturation, and value (lightness); R, G, and B are channels for RGB color space, representing red, green, and blue; NIR1 and NIR2 are the near-infrared channels of MS1 and MS2 cameras, respectively; Symbols '&', '∣', and '~' are logical operations in image processing (for MATLAB), representing 'And', 'Or', and 'Not', e.g. ~canopyMask means excluding canopy pixels (during flower detection).

Table S2. Correlation coefficient between yield and features extracted from image data or visual rating scores

Sensing method		Proximal (C-RGB)			Remote (D-RGB)			
**Flowering stage**		**Early**	**Mid**	**Late**		**Early**	**Mid**	**Late**
Winter canola	Visual rating	0.60 ***	0.74 ***	-0.43 ***	Flower area (15 m)	0.75 ***	0.84 ***	-0.18 *
	Flower area	0.65 ***	0.70 ***	-0.38 ***	Flower% (15 m)	0.53 ***	0.75 ***	-0.44 ***
	Flower%	0.51 ***	0.64 ***	-0.50 ***	Flower area (30 m)	0.72 ***	0.83 ***	-0.12 ns
					Flower% (30 m)	0.52 ***	0.74 ***	-0.36 ***
Spring canola	Visual rating	na	0.21 **	0.07 ns	Flower area (15 m)	na	0.26 ***	0.03 ns
	Flower area	na	0.21 **	0.05 ns	Flower% (15 m)	na	0.18 *	0.02 ns
	Flower%	na	0.20 *	0.05 ns	Flower area (30 m)	na	0.20 **	0.06 ns
					Flower% (30 m)	na	0.19 *	0.06 ns
Pea	Visual rating	-0.03 ns	0.09 ns	0.08 ns	Flower area (15 m)	na	0.22 **	0.25 **
	Flower area	0.00 ns	0.19 *	0.17 *	Flower% (15 m)	na	0.20 *	0.22 **
	Flower%	-0.01 ns	0.16 *	0.12 ns	Flower area (30 m)	na	0.15 ns	0.21 **
					Flower% (30 m)	na	0.14 ns	0.19 **
Chickpea	Visual rating	0.36 **	0.48 ***	0.10 ns	Flower area (15 m)	na	-0.24 ns	-0.33 **
	Flower area	0.33 **	0.37 **	0.36 **	Flower% (15 m)	na	-0.29 *	-0.36 **
	Flower%	0.03 ns	-0.02 ns	-0.02 ns	Flower area (30 m)	na	-0.30 *	-0.31 *
					Flower% (30 m)	na	-0.31 *	-0.32 *

Flower area: the area of flowers in terms of pixels; flowers% is the percentage of flowers, or the ratio of flower area to canopy area that includes flowers. na: not available. ns: statistically non-significant at the 0.05 probability level; *, **, and ***: statistically significant at 0.05, 0.01, and 0.001 probability levels, respectively.
